# Editorial: Smart Hospital Innovation: Technology, Service, and Policy

**DOI:** 10.3389/fpubh.2022.845577

**Published:** 2022-03-03

**Authors:** Hao Hu, Jinya Su, Jingdong Ma

**Affiliations:** ^1^State Key Laboratory of Quality Research in Chinese Medicine, Institute of Chinese Medical Sciences, University of Macau, Macao SAR, China; ^2^Department of Public Health and Medicinal Administration, Faculty of Health Sciences, University of Macau, Macao SAR, China; ^3^School of Computer Science and Electronic Engineering, University of Essex, Colchester, United Kingdom; ^4^School of Medicine and Health Management, Tongji Medical College of Huazhong University of Science and Technology, Wuhan, China

**Keywords:** healthcare technology, internet medical services, smart hospital innovation, internet hospital, information technology

Smart hospital is an emerging and fast-developing innovation in the context of a rapid increase of internet users, an increasing demand for high-quality medical resources, and an aging population worldwide. It generally involves the use of the optimized and automated processes built on an information and communications technology (ICT) environment, especially based on Internet of things (IoT), to offer assessment, consultation, direct treatment, services, and integrated care. This innovation has the potentials and already gained initial evidence to improve healthcare access, efficiency, or even effectiveness. Yet, along with these positive impacts and promises, a number of issues and challenges also appear. The aim of this Research Topic is to capture the current status of smart hospital innovation and to provide future directions in terms of technology, service, and policy. The Research Topic consists of 5 articles on smart hospital innovation including Original Research, Brief Research Report and Opinion.

In modern healthcare system, medical paper files have been replaced by patient Electronic Health Record (EHR) *via* keyboard-and-mouse interface. This transformation offered undisputable benefits, however, has also brought a clerical burden for the physicians. The authors in Misrai et al. contributed an opinion paper to discuss the burden of keyboard-and-mouse interface in EHR to physicians and the impairments to patient participation in medical care conversations. They also discussed the current status and prospects of more intuitive human-to-EHR interfaces (e.g., chat-bots) enabled by artificial intelligence and natural language processing. It was highlighted that although computer mice will no longer stand as the only human-to-EHR interface but still have a place to strive in the upcoming years: the operating room where surgeon will use it to control (non)semi-autonomous surgical robots.

Acceleration toward digital societies is creating unpredicted opportunities, however, at the same time is also potentially amplifying complex inequalities including how different communities access technologies, use them and benefit from them. The authors in Low et al. contributed a brief research paper to qualitatively (*via* in-depth semi-structured interview) investigated the attitudes and perceptions toward healthcare technology adoption among older adults in Singapore, where smart nation is a key initiative to move toward digitalization. It was discovered that the participants had a positive attitude toward healthcare technology, but they did not perceive an immediate need to adopt them. They also expressed an openness to adopt these technologies to maintain their health goals and use health service more efficiently, as long as affordability, personal data protection, and ease-of-use of the technologies were ensured. The study stressed the need to develop elderly with different attitudes and usage levels of technology, where practical recommendations to four personae were also made. Similarly, the authors in Tu et al. contributed a research paper to investigate older people's perceptions and experiences with Internet and Mobile Technology adoption (e.g., online appointment booking, hospital guidance, payment, and report checking) in hospitals by interviewing 29 older people at a tertiary hospital in Guangzhou, China. Qualitatively thematic analysis showed that various factors impact their readiness (e.g., major barriers and potential facilitators) for technology use, including their educational level, age, past experience, living arrangement, etc. Therefore, policy design should consider the diversified experiences and general problems that older people encountered and introduce targeted measures accordingly to facilitate older people's technology adoption.

Cancer management has been a heavy burden on the global healthcare systems, particularly with an increasing cancer population worldwide. The authors in Dai et al. investigated the current situation of the out-of-hospital management of patients with cancer and evaluate the feasibility of internet medical intervention outside the hospital in China. Analysis results of 1,171 qualified questionnaires in 13 large-scale hospitals in Sichuan Province, China showed that 92.7% of patients with cancer experienced varying degrees of out-of-hospital symptoms after treatment, and a third of them needed clinical intervention. Among them, 92% of respondents required medical help outside the hospital, and 65% were willing to pay for the out-of-hospital management. More than half of the life or work of patients with cancer are still greatly affected under the current management model and therefore out-of-hospital management needs to be improved in China.

The population of Chinese physicians is frequently threatened by abnormal death, including death by overwork or homicide, which is not only a health problem but also a social problem. Therefore, the authors in Liang et al. analyzed the characteristics of abnormal death of physicians in Chinese hospitals from 2007 to 2020, and investigated the relationship between abnormal death and physician workload. The analysis revealed that there was a strong correlation between the number of abnormal deaths of physicians in China and the number of inpatients per physician (*r* = 0.683, *P* = 0.01). It was suggested that smart hospital technologies have the potential to alleviate this situation by reducing physicians' workload.

With the continuous efforts in this field, it is expected that smart hospital could be a driving force to improve the quality and effectiveness of health care in the coming future.

## Author Contributions

JS drafted the manuscript. All authors listed have reviewed this work and approved its publication.

## Conflict of Interest

The authors declare that the research was conducted in the absence of any commercial or financial relationships that could be construed as a potential conflict of interest.

## Publisher's Note

All claims expressed in this article are solely those of the authors and do not necessarily represent those of their affiliated organizations, or those of the publisher, the editors and the reviewers. Any product that may be evaluated in this article, or claim that may be made by its manufacturer, is not guaranteed or endorsed by the publisher.

